# A primary squamous cell carcinoma of the sigmoid colon in a young patient: A case report and literature review

**DOI:** 10.1002/ccr3.6194

**Published:** 2022-08-16

**Authors:** Mohanad Faisal, Ahmed Abdalhadi, M. Zaki Karzoun, Mohamed Aboagla Suliman Mohamed, Mouhammad Zuhair Sharaf Elden, Alaaeldin Shablak

**Affiliations:** ^1^ Department of Medical Oncology, National Center for Cancer Care and Research Hamad Medical Corporation Doha Qatar; ^2^ Department of Laboratory Medicine and Pathology Hamad Medical Corporation Doha Qatar

**Keywords:** colon, oncology, pathology, squamous cell carcinoma, squamous cell carcinomacolon cancer

## Abstract

Colon cancer is among the most common types of cancer with adenocarcinomas being the most common type. Herein we report a young patient who presented with primary colonic squamous cell carcinoma without risk factors. To the best of our knowledge, this is the youngest patient with such diagnosis worldwide.

## Graphical abstract

We reported a case of colon SCC in young patient to increase the awareness about this rare malignancy among oncologists with quick review about pathophysiology and management.

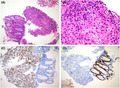

## 1 INTRODUCTION

More than 90% of colorectal carcinomas are adenocarcinomas originating from epithelial cells of the colorectal mucosa.^1^ Other rare types of colorectal carcinomas include neuroendocrine, squamous cell, adeno‐squamous, spindle cell, and undifferentiated carcinomas. Conventional adenocarcinoma is characterized by glandular formation, which is the basis for histological tumor grading into well, moderately, and poorly differentiated adenocarcinoma.^2^ The histopathological classification of colorectal cancer carries important prognostic information^3^ and provides important information on the carcinogenesis pathway.^4^ On the other hand, primary squamous cell carcinoma (SCC) and adeno‐squamous cell carcinoma of the colon are uncommon histological types and their characteristics are less well known.^5^ It's usually diagnosed after the age of 50 and can be presented at any site and is associated with poor prognosis.^6^ Approximately 150 cases of colorectal SCC have been reported, with the majority arising from the rectum, followed by the right colon.^7^

Colorectal SCCs tend to be more frequently locally invasive and more likely to involve regional lymph nodes than adenocarcinomas, probably due to delayed diagnosis.^8^

The mainstay of treatment for colon SCC is surgical resection. While there are some reports of patients with advanced disease who responded to systemic chemotherapy,^9^ the prognosis remains poor.

## 2 CASE PRESENTATION

A 38‐year‐old male Bangladeshi patient with no known chronic illness presented with left lower abdominal pain, fever, and loose stool in addition to significant weight loss. The stool frequency was around three times per day, non‐bloody and with little mucus. He is an ex‐smoker, nonalcoholic, and has no family history of cancer. He was febrile and other vitals were within normal limits. The physical examination revealed tenderness in the left lower quadrant. After a computed tomography scan (CT) of the abdomen, the patient was initially diagnosed with sigmoid diverticulitis with a pericolic abscess. He was managed conservatively with antibiotics, bowel rest, and intravenous (IV) fluid with the plan to do colonoscopy as an outpatient.

**FIGURE 1:**
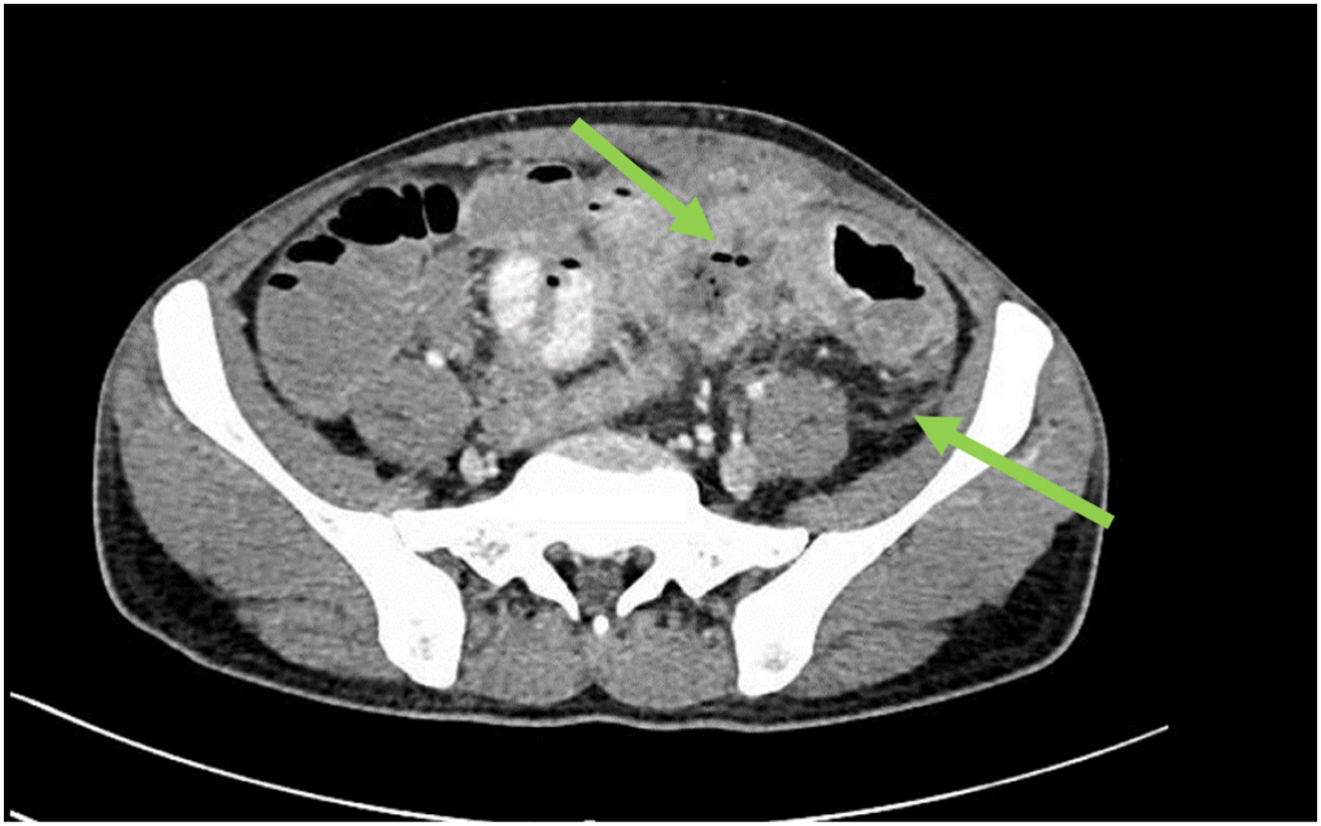
Abdominal CT scan showed thickened sigmoid colon with abdominal lymphadenopathy (arrows).

The patient presented again after a few weeks with similar symptoms of lower abdominal pain and diarrhea. A repeated CT scan of the abdomen showed a thickened sigmoid colon with abdominal lymphadenopathy in addition to evidence of colitis (Figure 1). Laboratory tests are shown in Table 1.

**TABLE 1:** Summary of laboratory tests on the patient's second admission

Laboratory test	Results	Normal ranges
Hemoglobin (gram/deciliter)	12.1	12.5–16
WBC/microliter	11,500	5000–1100
Platelet/microliter	494,000	150,000–450,000
Creatinine (micromole/liter)	66	65.4–119.3
ALT (unit/liter)	25	7–50
CEA (microgram/liter)	4.2	<2.5
CA19‐9(unit/milliliter)	13.3	0–37
AST (unit/liter)	19	10–50

**FIGURE 2:**
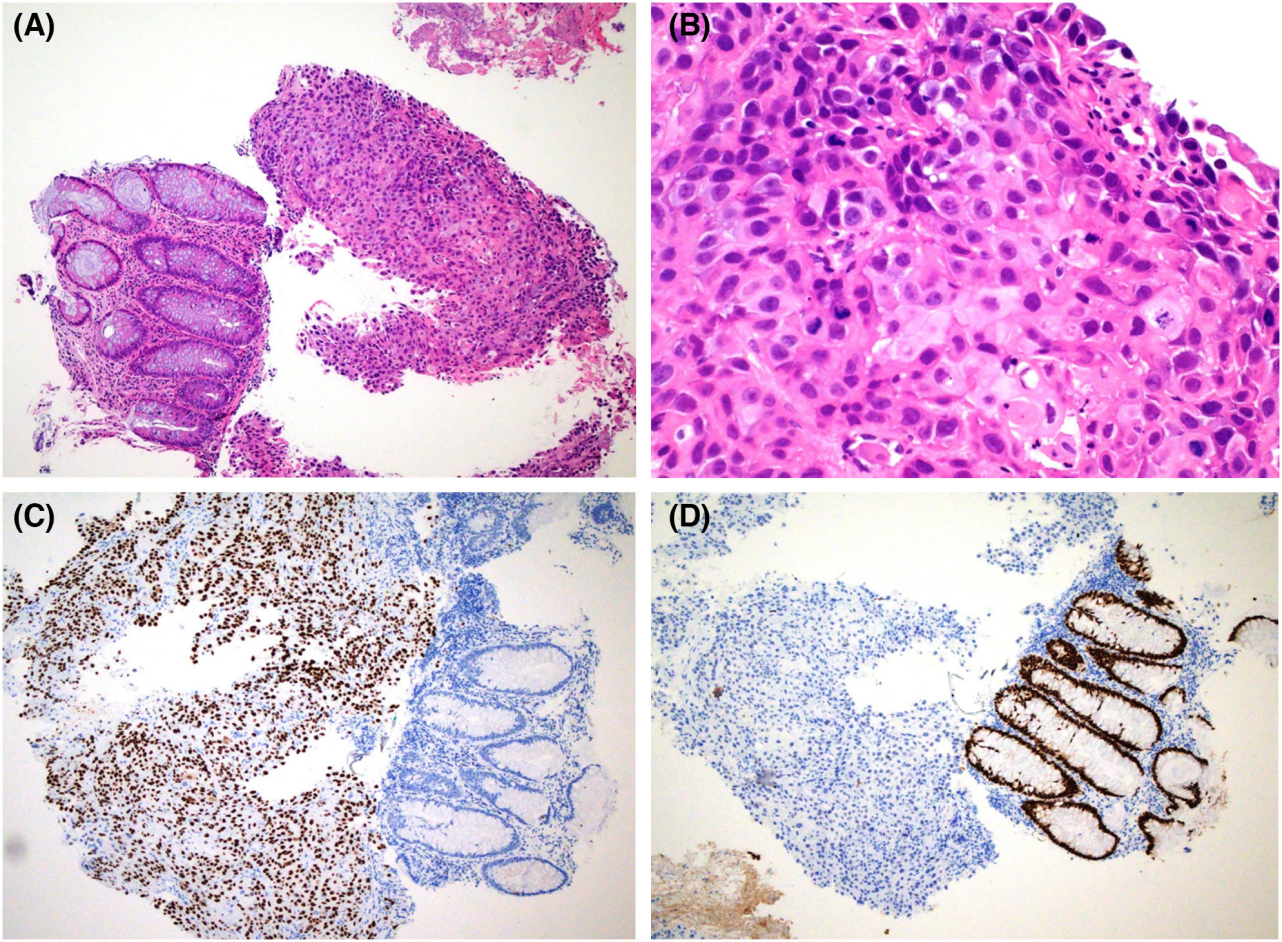
(A) The colonic mucosa is intact (left side of the field). The submucosa (right side) is infiltrated by sheets of neoplastic cells (4× magnification). (B) The neoplastic cells are atypical with distinct cell membrane and increased nuclear‐to‐cytoplasmic ratio. Miotic figures and apoptotic bodies are evident (40X magnification). (C) p63 immunohistochemical stain. The neoplastic cells (on the left side of the field) are positive. (D) CDX2 immunohistochemical stain is negative in the tumor cells (left side of the field), while positive in the normal colonic mucosa.

Upon infection resolution, the patient underwent colonoscopy and was found to have a large, ulcerated, obstructing mass in the sigmoid colon, and a biopsy was taken. Histopathology sections (Figure 2) showed normal colonic mucosa with infiltration of the submucosa by sheets of polygonal epithelioid cells with round nuclei, clumped chromatin, abundant eosinophilic cytoplasm, distinct cell membrane, and intercellular bridges. Multiple mitoses and apoptotic bodies are identified. By immunohistochemical stains, the tumor cells were positive for the squamous markers p63 and p40. They were negative for CDX2, CK20, SATB2, CK7, PAX8, GATA3, TTF1, synaptophysin, chromogranin, SALL4, OCT3/4, Melan‐A, and SOX 10.

Mismatch repair (MMR) immunohistochemical stains showed loss of mismatch repair protein MutS homolog 2 (MSH2) with retained mutS homolog 6 (MSH6), MutL homolog 1 (MLH1), and Mismatch repair endonuclease (PMS2). Mutation of rat sarcoma viral oncogenes (KRAS, PRAS, NRAS) and v‐raf murine sarcoma viral oncogene homolog B1 (BRAF) were not detected.

**FIGURE 3:**
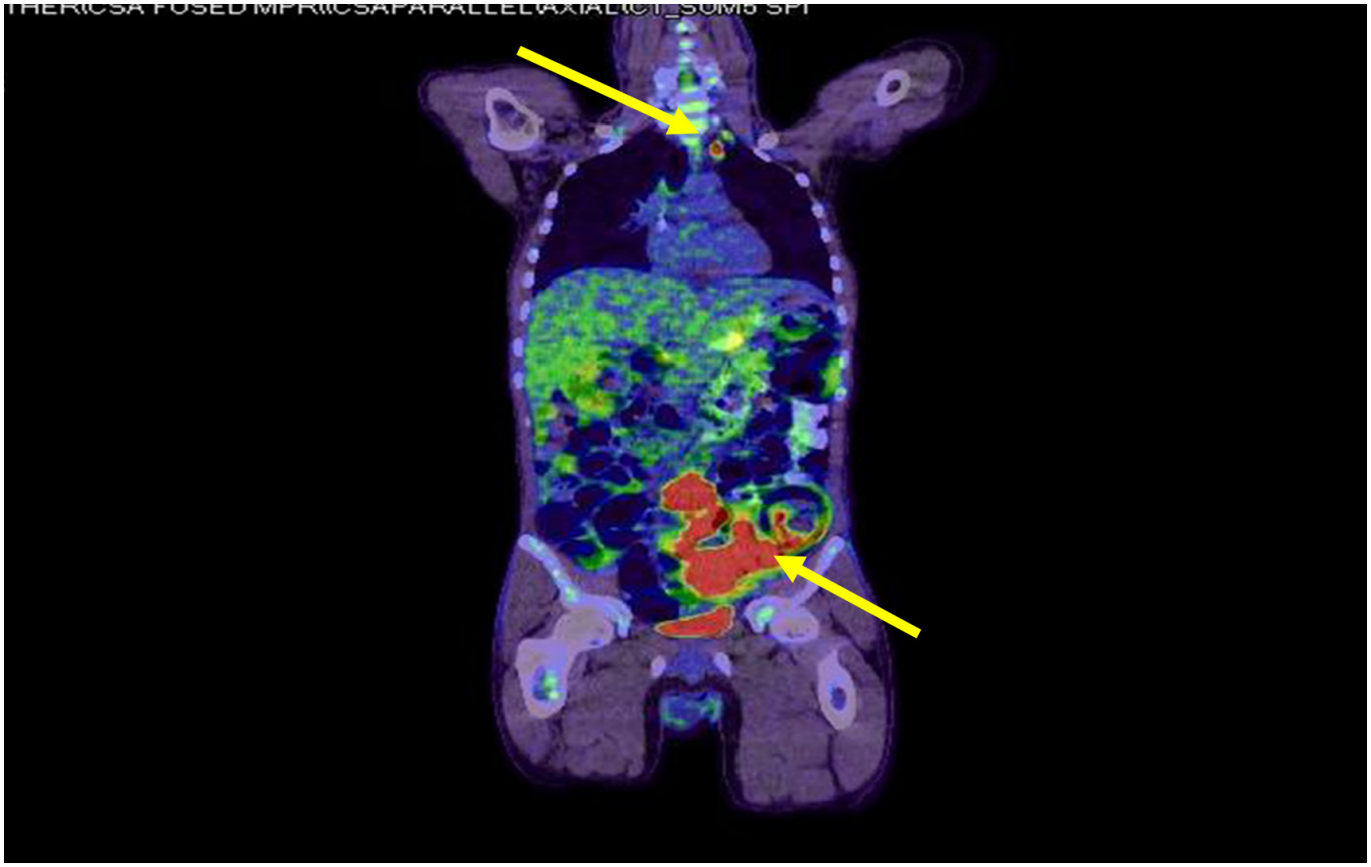
PET/CT showed a thickening of the distal sigmoid colon and distant lymph nodes in the left supraclavicular region.

Positron emission tomography (PET) showed a thickening of the distal sigmoid colon with local invasion of the anterior abdominal wall in addition to involvement of locoregional and distant lymph nodes in the left supraclavicular region (Figure 3).

During the hospital admission course, he developed bowel obstruction, and laparotomy with transverse colostomy was performed.

After a colorectal multidisciplinary team (MDT) meeting, the diagnosis of stage 4 SCC of the sigmoid colon was established and the patient was planned to be started on palliative chemotherapy in view of borderline performance status (The Eastern Cooperative Oncology Group performance status: 3). The patient received the FOLFOX chemotherapy regimen (folinic acid, fluorouracil, and oxaliplatin). After receiving the first cycle, the patient requested to travel to his home country, and he declined further chemo or immunotherapy.

## 3 DISCUSSION

Here we discuss the case of a young Asian male who presented with stage 4 squamous cell carcinoma complicated by intestinal obstruction, who was managed by a diverting colostomy and given one cycle of chemotherapy before declining further care.

Squamous cell carcinoma usually affects the skin, lungs, and upper gastrointestinal tract, but other rare sites have been reported.^10^ The first case of colonic SCC reported in the literature was in 1919, by Schmidtian.^11^ It usually occurs in patients above the age of 50 years, with a median age of 56.9 ± 12.19 years (mean ± SD) as found in one study.^12^ Although SCC of the rectum has been reported under the age of 40,^13^ cases of colon SCC in people under the age of 40 have not been reported, which makes our patient the first reported case at this age according to our search.

The pathogenesis of colon SCC is not fully understood. Several theories have been discussed for the carcinogenesis of colorectal SCC. The presence of pluripotent stem cells of endodermal origin in the colonic mucosa can differentiate into squamous epithelium that could undergo malignant transformation.^14^ Another hypothesis includes damage to the colonic mucosa, which could result in proliferation of basal cells into squamous cells.^5^ Such damage can be caused by irritation, inflammatory bowel disease, infection, exposure to asbestos and radiation, which may result in squamous cell metaplasia of colorectal epithelium.^9^, ^14^, ^15^ The presence of Human Papilloma Virus (HPV) has been hypothesized to be possibly associated with colorectal SCC, as it damages local cell proliferation, thus inducing oncogenesis,^16^ but the association is not fully proved yet. Indeed, our patient did not have any obvious etiology such as inflammatory conditions, evidence of HPV infection, or a family history of cancer to explain the pathogenesis of his cancer.

Immunohistochemistry has proved useful in the diagnosis of primary colorectal SCC and in the differentiation of squamous cell carcinomas of other primary sources.^13^ Immunohistochemistry is also helpful in giving prognostic information depending on the presence of certain biomarkers.^17^

Colorectal SCC is associated with a poor prognosis. One study showed a 5‐year overall survival rate of 50% for Duke B carcinomas, 33% for Duke C carcinomas, and for Duke D is 0%.^18^ Another review showed 1‐year cumulative survival reached 81.2%, while 5‐year survival was 49.5%.^12^

Treatment of colon SCC mainly involves surgical excision of the tumor when possible.^13^, ^18^ The chemotherapy regimen along with the surgery or as primary treatment for metastatic disease is yet to be determined. Treatment regimens like those applied for anal SCC that involve 5‐fluorouracil‐based treatments in combination with mitomycin‐C or cisplatin have been explored with some success.^13^ Moreover, the role of radiotherapy in the colon SCC is unclear, although some consider it as primary treatment along with chemotherapy in rectal SCC.^19^, ^20^ With the view of the response of colon cancer with microsatellite instability (MSI) to immunotherapy,^21^, ^22^ we offered immunotherapy to our patient as he is (MSH2) deficient. He received one cycle of FOLFOX and declined further treatments and was repatriated home.

## 4 CONCLUSION

Squamous cell carcinoma of the colon is a very rare entity with no clear pathogenesis and its management is still evolving. Further studies to define the best ways to manage such cases are needed.

## AUTHOR CONTRIBUTIONS

Mohanad Faisal: manuscript writing, literature review, consent, follow up, submission. Ahmed Abdalhadi: manuscript writing, clinical follow‐up. M. Zaki Karzoun: pathology reports and slides. Mohamed Aboagla Suliman mohamed: manuscript writing, clinical follow‐up. Mouhammad Zuhair Sharaf Eldean: pathology reports and slides. Alaaeldin Shablak: manuscript writing, clinical follow‐up, supervision.

## CONFLICT OF INTEREST

The authors declare that there is no conflict of interest in the publication of this article.

## CONSENT

Written informed consent was obtained from the patient to publish this report in accordance with the journal's patient consent policy.

## Data Availability

The data that support the findings of this study are available from the corresponding author upon reasonable request.
